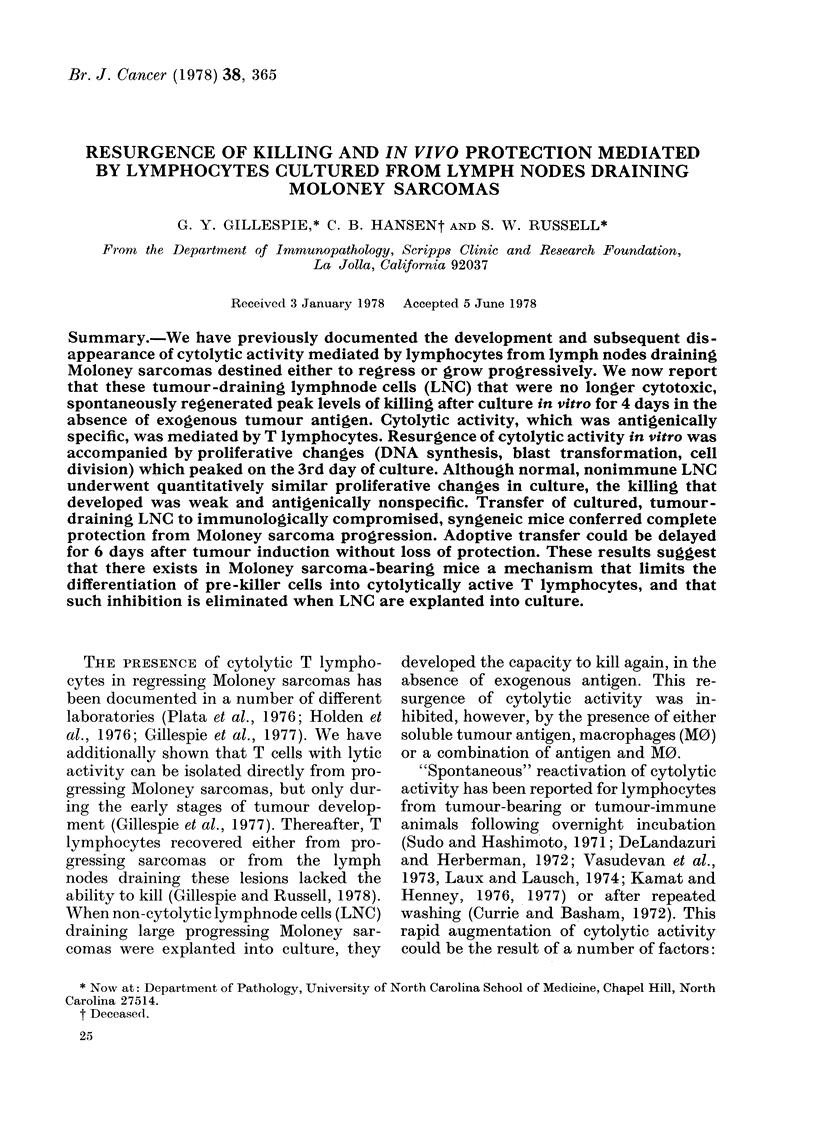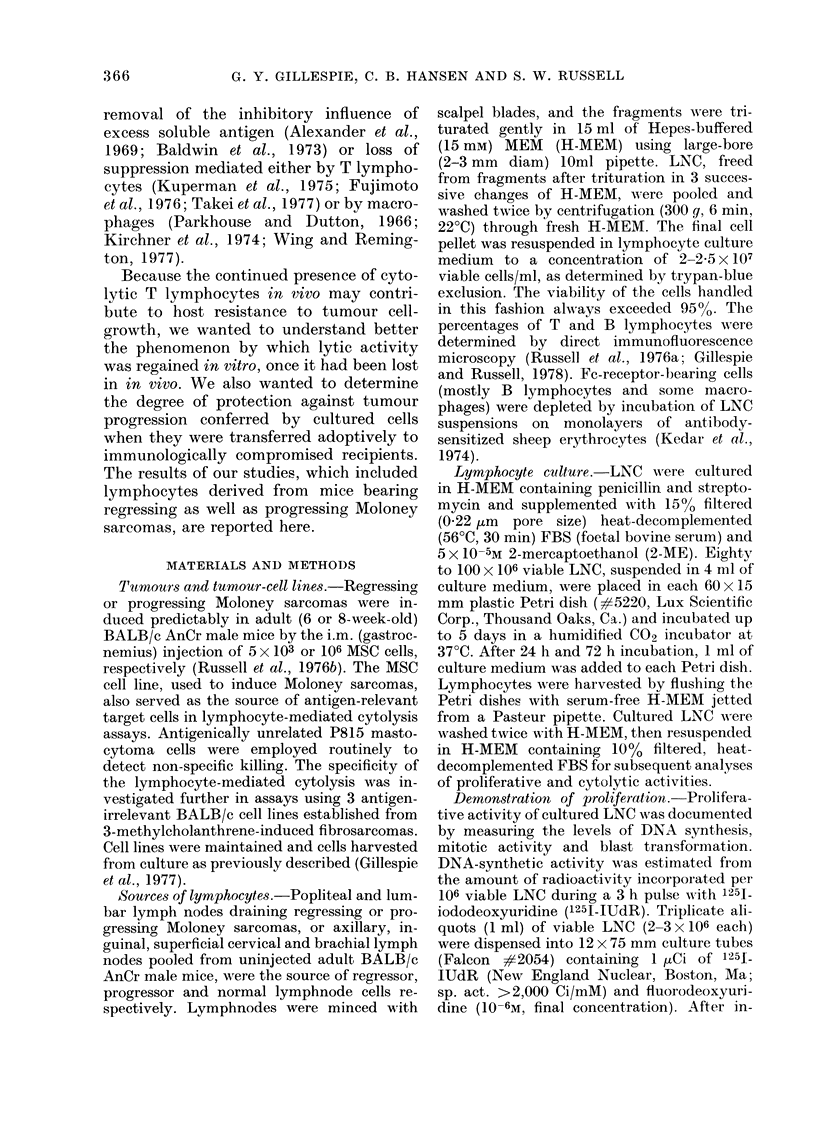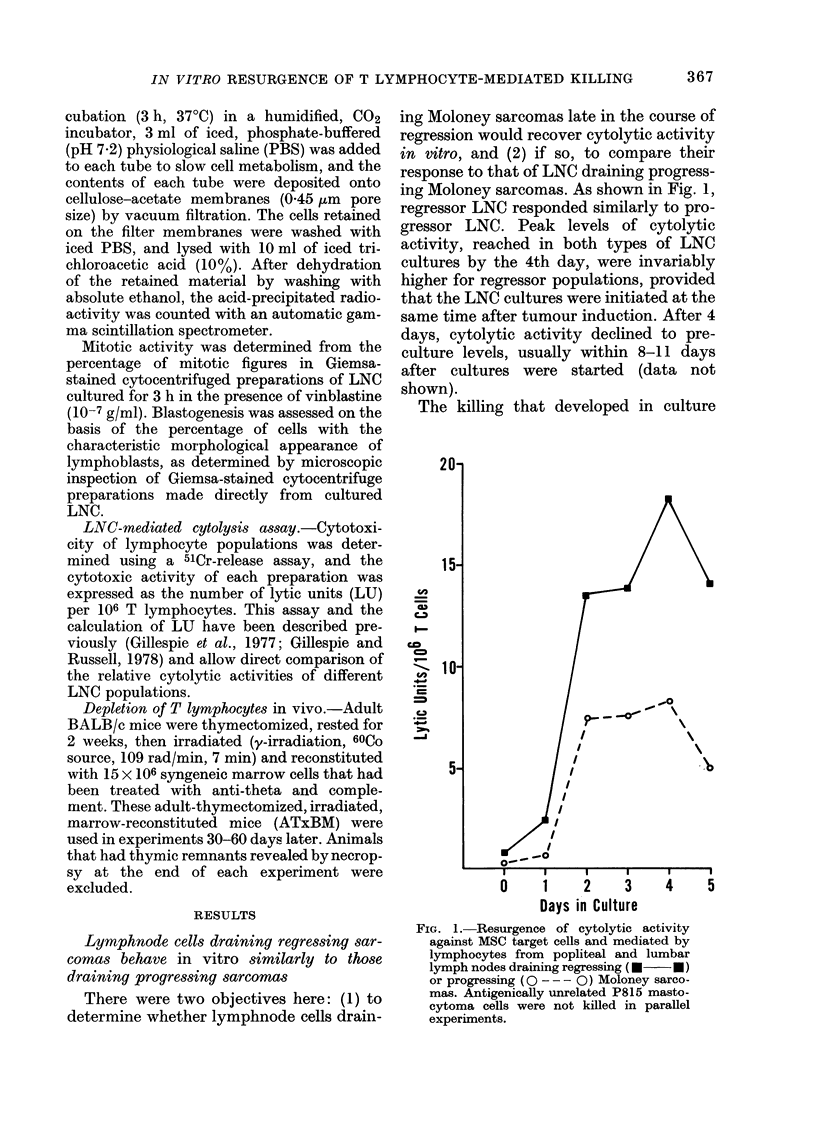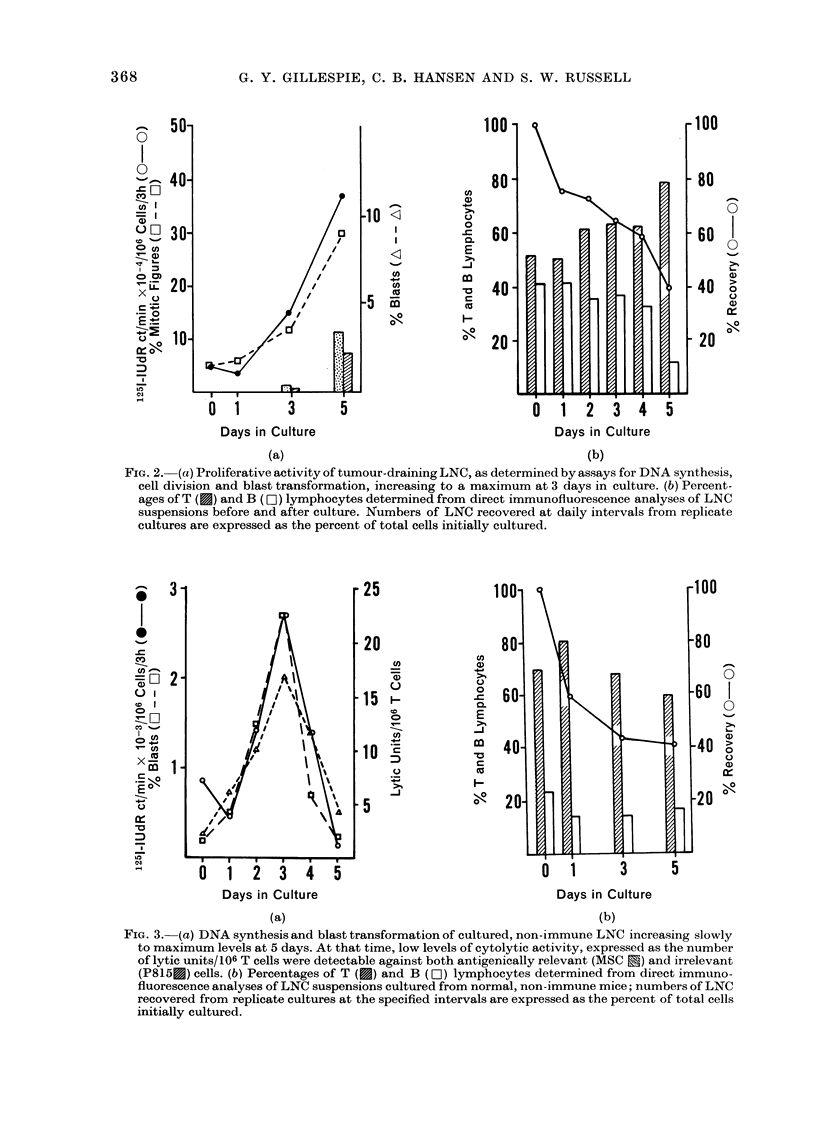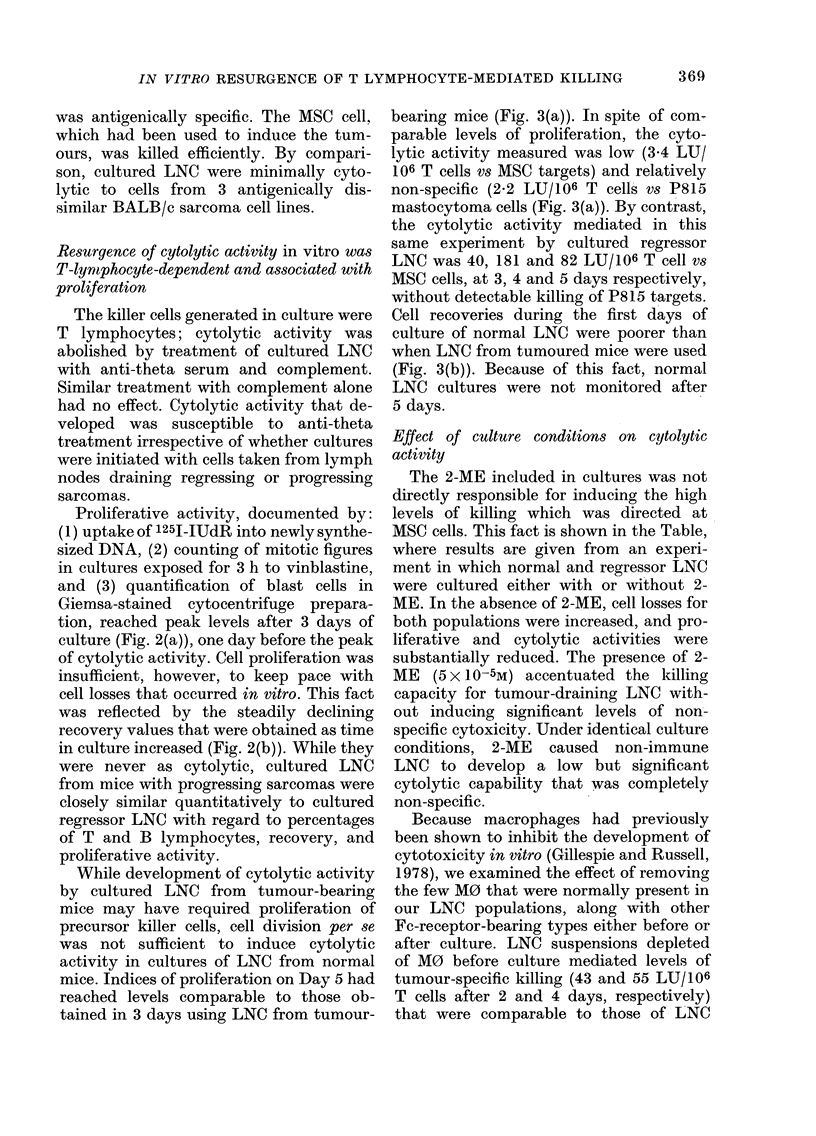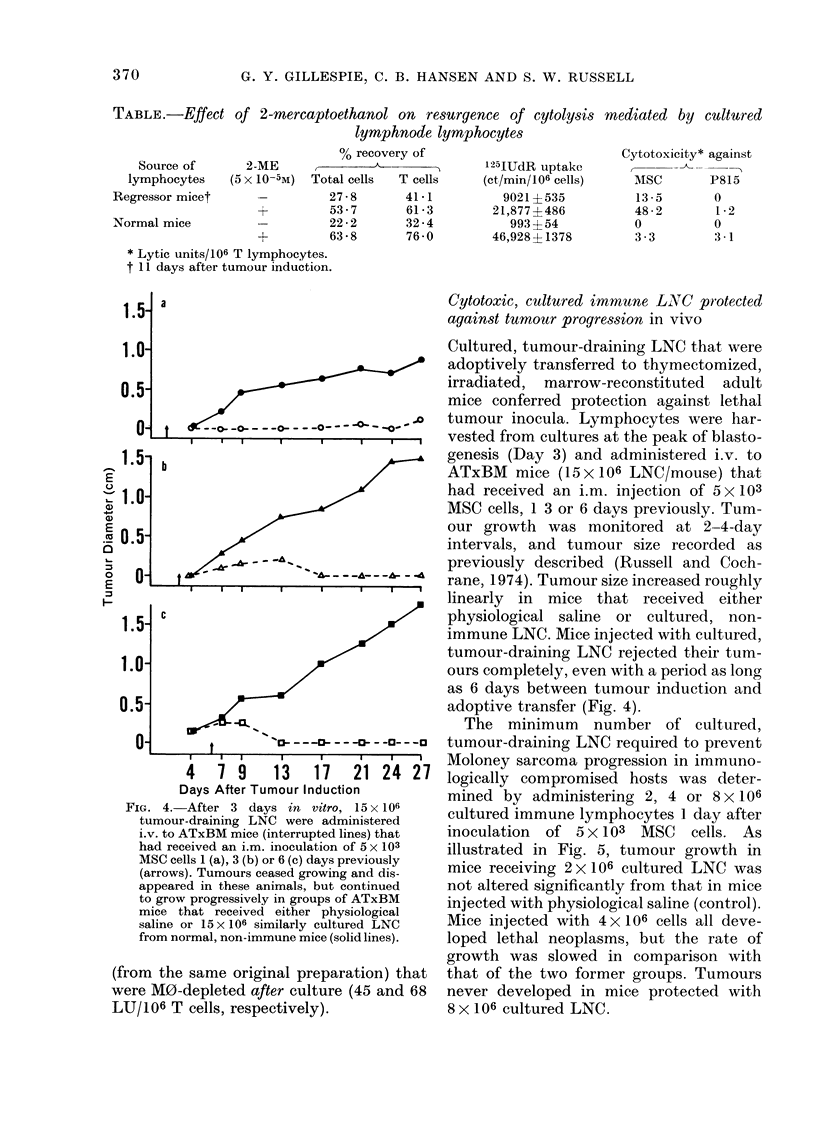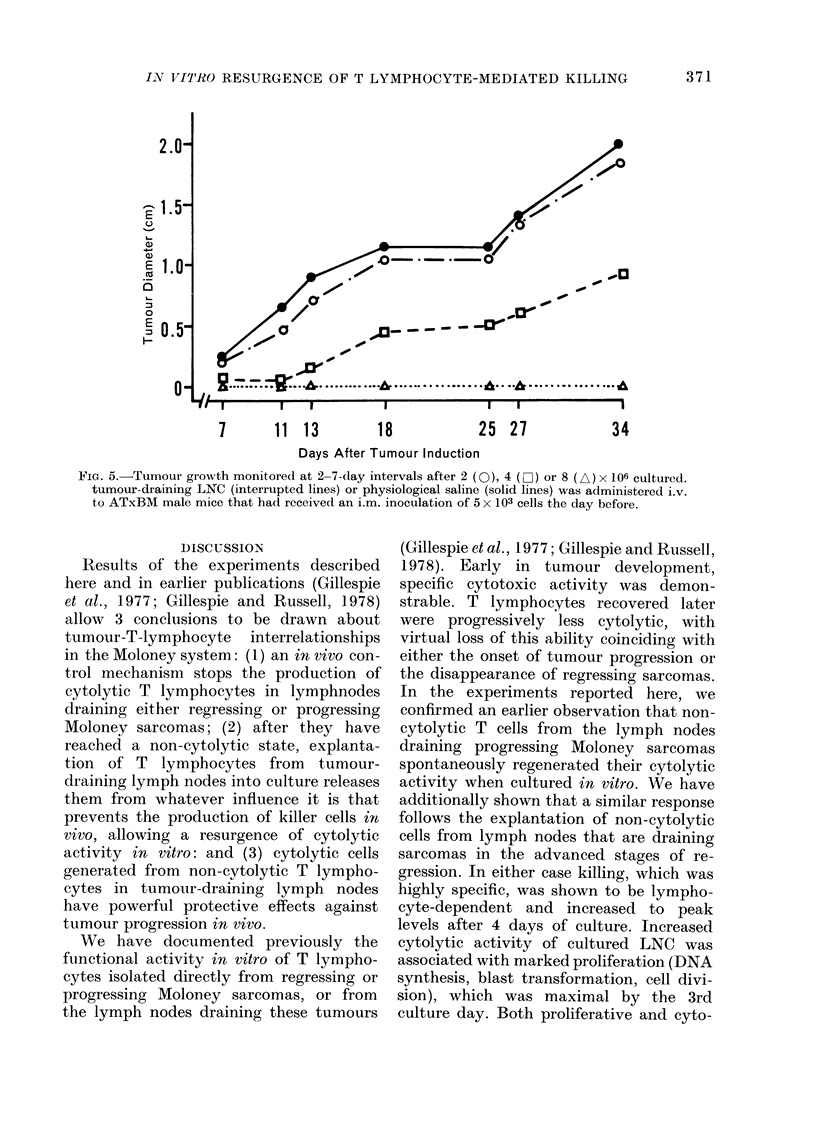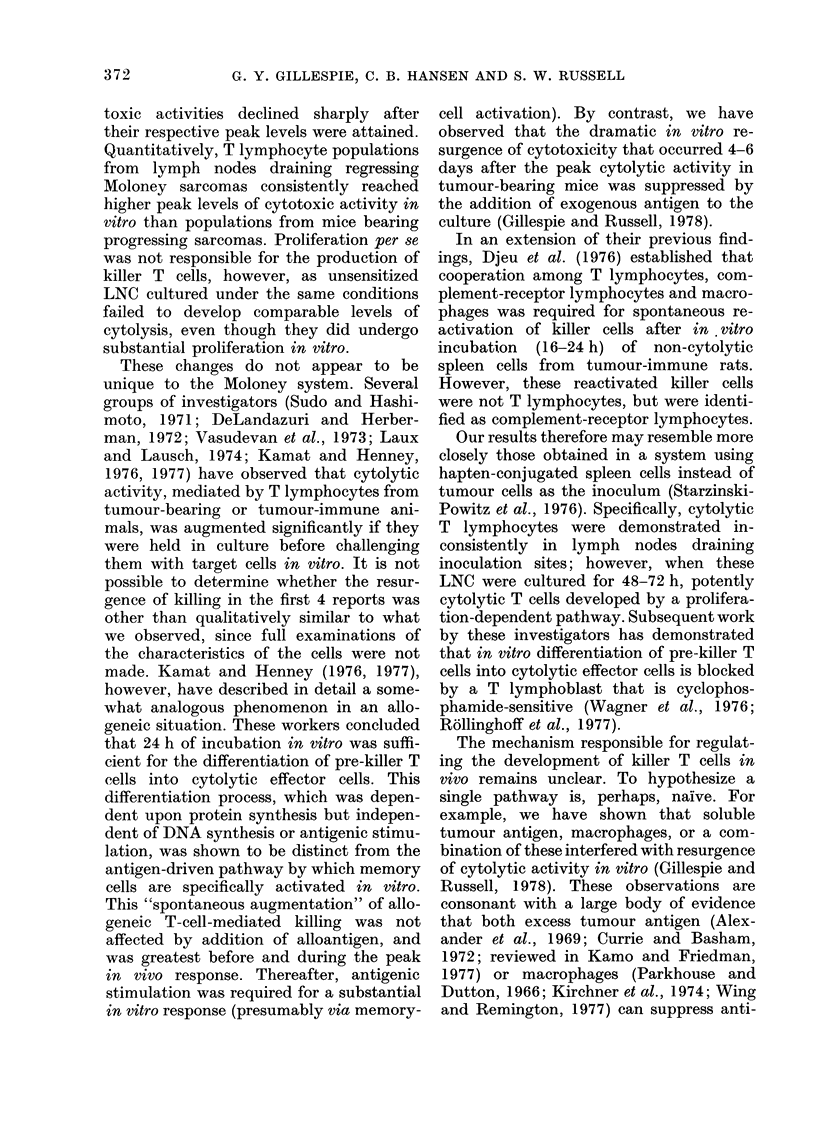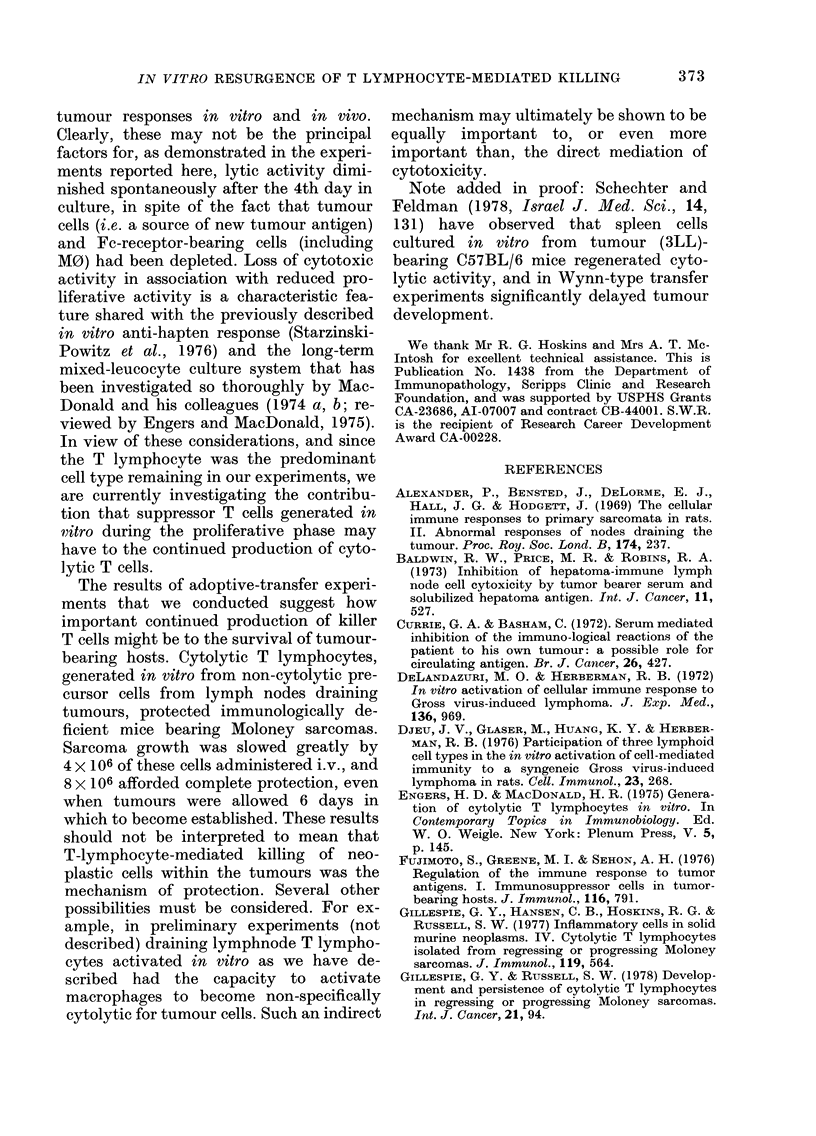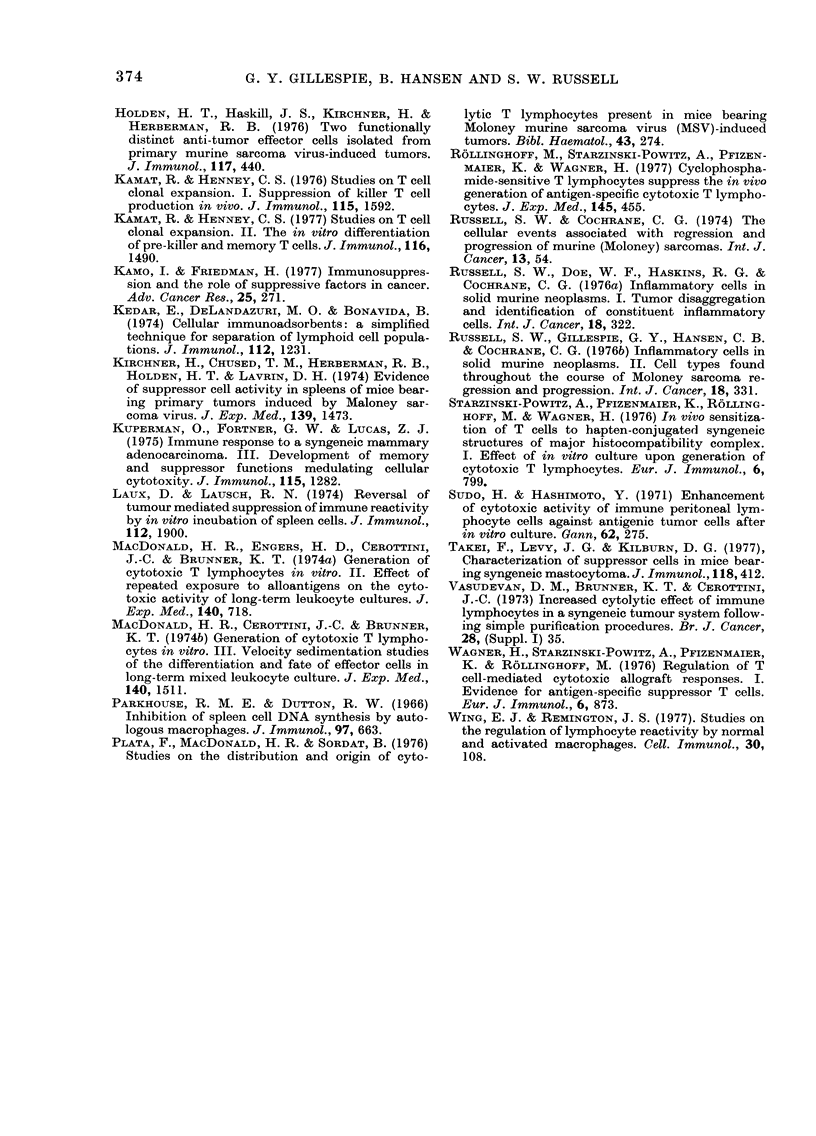# Resurgence of killing and in vivo protection mediated by lymphocytes cultured from lymph nodes draining Moloney sarcomas.

**DOI:** 10.1038/bjc.1978.216

**Published:** 1978-09

**Authors:** G. Y. Gillespie, C. B. Hansen, S. W. Russell

## Abstract

We have previously documented the development and subsequent disappearance of cytolytic activity mediated by lymphocytes from lymph nodes draining Moloney sarcomas destined either to regress or grow progressively. We now report that these tumour-draining lymphnode cells (LNC) that were no longer cytotoxic, spontaneously regenerated peak levels of killing after culture in vitro for 4 days in the absence of exogenous tumour antigen. Cytolytic activity, which was antigenically specific, was mediated by T lymphocytes. Resurgence of cytolytic activity in vitro was accompanied by proliferative changes (DNA synthesis, blast transformation, cell division) which peaked on the 3rd day of culture. Although normal, nonimmune LNC underwent quantitatively similar proliferative changes in culture, the killing that developed was weak and antigenically nonspecific. Transfer of cultured, tumour-draining LNC to immunologically compromised, syngeneic mice conferred complete protection from Moloney sarcoma progression. Adoptive transfer could be delayed for 6 days after tumour induction without loss of protection. These results suggest that there exists in Moloney sarcoma-bearing mice a mechanism that limits the differentiation of pre-killer cells into cytolytically active T lymphocytes, and that such inhibition is eliminated when LNC are explanted into culture.


*Br. J. Cancer* (1978) **38**, 365

### RESURGENCE OF KILLING AND *IN VIVO* PROTECTION MEDIATED BY LYMPHOCYTES CULTURED FROM LYMPH NODES DRAINING MOLONEY SARCOMAS

G. Y. GILLESPIE,* C. B. HANSEN† AND S. W. RUSSELL*

*From the Department of Immunopathology, Scripps Clinic and Research Foundation, La Jolla, California* 92037

Received 3 January 1978 Accepted 5 June 1978

**Summary.—We have previously documented the development and subsequent disappearance of cytolytic activity mediated by lymphocytes from lymph nodes draining Moloney sarcomas destined either to regress or grow progressively. We now report that these tumour-draining lymphnode cells (LNC) that were no longer cytotoxic, spontaneously regenerated peak levels of killing after culture *in vitro* for 4 days in the absence of exogenous tumour antigen. Cytolytic activity, which was antigenically specific, was mediated by T lymphocytes. Resurgence of cytolytic activity *in vitro* was accompanied by proliferative changes (DNA synthesis, blast transformation, cell division) which peaked on the 3rd day of culture. Although normal, nonimmune LNC underwent quantitatively similar proliferative changes in culture, the killing that developed was weak and antigenically nonspecific. Transfer of cultured, tumour-draining LNC to immunologically compromised, syngeneic mice conferred complete protection from Moloney sarcoma progression. Adoptive transfer could be delayed for 6 days after tumour induction without loss of protection. These results suggest that there exists in Moloney sarcoma-bearing mice a mechanism that limits the differentiation of pre-killer cells into cytolytically active T lymphocytes, and that such inhibition is eliminated when LNC are explanted into culture.**

THE PRESENCE of cytolytic T lymphocytes in regressing Moloney sarcomas has been documented in a number of different laboratories (Plata *et al.*, 1976; Holden *et al.*, 1976; Gillespie *et al.*, 1977). We have additionally shown that T cells with lytic activity can be isolated directly from progressing Moloney sarcomas, but only during the early stages of tumour development (Gillespie *et al.*, 1977). Thereafter, T lymphocytes recovered either from progressing sarcomas or from the lymph nodes draining these lesions lacked the ability to kill (Gillespie and Russell, 1978). When non-cytolytic lymphnode cells (LNC) draining large progressing Moloney sarcomas were explanted into culture, they developed the capacity to kill again, in the absence of exogenous antigen. This resurgence of cytolytic activity was inhibited, however, by the presence of either soluble tumour antigen, macrophages (MØ) or a combination of antigen and MØ.

"Spontaneous" reactivation of cytolytic activity has been reported for lymphocytes from tumour-bearing or tumour-immune animals following overnight incubation (Sudo and Hashimoto, 1971; DeLandazuri and Herberman, 1972; Vasudevan *et al.*, 1973, Laux and Lausch, 1974; Kamat and Henney, 1976, 1977) or after repeated washing (Currie and Basham, 1972). This rapid augmentation of cytolytic activity could be the result of a number of factors:

* Now at: Department of Pathology, University of North Carolina School of Medicine, Chapel Hill, North Carolina 27514.

† Deceased.

removal of the inhibitory influence of excess soluble antigen (Alexander *et al.*, 1969; Baldwin *et al.*, 1973) or loss of suppression mediated either by T lymphocytes (Kuperman *et al.*, 1975; Fujimoto *et al.*, 1976; Takei *et al.*, 1977) or by macrophages (Parkhouse and Dutton, 1966; Kirchner *et al.*, 1974; Wing and Remington, 1977).

Because the continued presence of cytolytic T lymphocytes *in vivo* may contribute to host resistance to tumour cell-growth, we wanted to understand better the phenomenon by which lytic activity was regained *in vitro*, once it had been lost in *in vivo*. We also wanted to determine the degree of protection against tumour progression conferred by cultured cells when they were transferred adoptively to immunologically compromised recipients. The results of our studies, which included lymphocytes derived from mice bearing regressing as well as progressing Moloney sarcomas, are reported here.

### MATERIALS AND METHODS

*Tumours and tumour-cell lines.*—Regressing or progressing Moloney sarcomas were induced predictably in adult (6 or 8-week-old) BALB/c AnCr male mice by the i.m. (gastrocnemius) injection of $5 \times 10^3$ or $10^6$ MSC cells, respectively (Russell *et al.*, 1976*b*). The MSC cell line, used to induce Moloney sarcomas, also served as the source of antigen-relevant target cells in lymphocyte-mediated cytolysis assays. Antigenically unrelated P815 mastocytoma cells were employed routinely to detect non-specific killing. The specificity of the lymphocyte-mediated cytolysis was investigated further in assays using 3 antigen-irrelevant BALB/c cell lines established from 3-methylcholanthrene-induced fibrosarcomas. Cell lines were maintained and cells harvested from culture as previously described (Gillespie *et al.*, 1977).

*Sources of lymphocytes.*—Popliteal and lumbar lymph nodes draining regressing or progressing Moloney sarcomas, or axillary, inguinal, superficial cervical and brachial lymph nodes pooled from uninjected adult BALB/c AnCr male mice, were the source of regressor, progressor and normal lymphnode cells respectively. Lymphnodes were minced with scalpel blades, and the fragments were triturated gently in 15 ml of Hepes-buffered (15 mM) MEM (H-MEM) using large-bore (2–3 mm diam) 10ml pipette. LNC, freed from fragments after trituration in 3 successive changes of H-MEM, were pooled and washed twice by centrifugation (300 *g*, 6 min, 22°C) through fresh H-MEM. The final cell pellet was resuspended in lymphocyte culture medium to a concentration of $2–2{\cdot}5 \times 10^7$ viable cells/ml, as determined by trypan-blue exclusion. The viability of the cells handled in this fashion always exceeded 95%. The percentages of T and B lymphocytes were determined by direct immunofluorescence microscopy (Russell *et al.*, 1976a; Gillespie and Russell, 1978). Fc-receptor-bearing cells (mostly B lymphocytes and some macrophages) were depleted by incubation of LNC suspensions on monolayers of antibody-sensitized sheep erythrocytes (Kedar *et al.*, 1974).

*Lymphocyte culture.*—LNC were cultured in H-MEM containing penicillin and streptomycin and supplemented with 15% filtered (0·22 $\mu$m pore size) heat-decomplemented (56°C, 30 min) FBS (foetal bovine serum) and $5 \times 10^{-5}$M 2-mercaptoethanol (2-ME). Eighty to $100 \times 10^6$ viable LNC, suspended in 4 ml of culture medium, were placed in each $60 \times 15$ mm plastic Petri dish (#5220, Lux Scientific Corp., Thousand Oaks, Ca.) and incubated up to 5 days in a humidified $CO_2$ incubator at 37°C. After 24 h and 72 h incubation, 1 ml of culture medium was added to each Petri dish. Lymphocytes were harvested by flushing the Petri dishes with serum-free H-MEM jetted from a Pasteur pipette. Cultured LNC were washed twice with H-MEM, then resuspended in H-MEM containing 10% filtered, heat-decomplemented FBS for subsequent analyses of proliferative and cytolytic activities.

*Demonstration of proliferation.*—Proliferative activity of cultured LNC was documented by measuring the levels of DNA synthesis, mitotic activity and blast transformation. DNA-synthetic activity was estimated from the amount of radioactivity incorporated per $10^6$ viable LNC during a 3 h pulse with $^{125}$I-iododeoxyuridine ($^{125}$I-IUdR). Triplicate aliquots (1 ml) of viable LNC ($2–3 \times 10^6$ each) were dispensed into $12 \times 75$ mm culture tubes (Falcon #2054) containing 1 $\mu$Ci of $^{125}$I-IUdR (New England Nuclear, Boston, Ma; sp. act. >2,000 Ci/mM) and fluorodeoxyuridine ($10^{-6}$M, final concentration). After incubation (3 h, 37°C) in a humidified, $CO_2$ incubator, 3 ml of iced, phosphate-buffered (pH 7·2) physiological saline (PBS) was added to each tube to slow cell metabolism, and the contents of each tube were deposited onto cellulose–acetate membranes (0·45 $\mu$m pore size) by vacuum filtration. The cells retained on the filter membranes were washed with iced PBS, and lysed with 10 ml of iced trichloroacetic acid (10%). After dehydration of the retained material by washing with absolute ethanol, the acid-precipitated radioactivity was counted with an automatic gamma scintillation spectrometer.

Mitotic activity was determined from the percentage of mitotic figures in Giemsa-stained cytocentrifuged preparations of LNC cultured for 3 h in the presence of vinblastine ($10^{-7}$ g/ml). Blastogenesis was assessed on the basis of the percentage of cells with the characteristic morphological appearance of lymphoblasts, as determined by microscopic inspection of Giemsa-stained cytocentrifuge preparations made directly from cultured LNC.

*LNC-mediated cytolysis assay.*—Cytotoxicity of lymphocyte populations was determined using a $^{51}$Cr-release assay, and the cytotoxic activity of each preparation was expressed as the number of lytic units (LU) per $10^6$ T lymphocytes. This assay and the calculation of LU have been described previously (Gillespie *et al.*, 1977; Gillespie and Russell, 1978) and allow direct comparison of the relative cytolytic activities of different LNC populations.

*Depletion of T lymphocytes* in vivo.—Adult BALB/c mice were thymectomized, rested for 2 weeks, then irradiated ($\gamma$-irradiation, $^{60}$Co source, 109 rad/min, 7 min) and reconstituted with $15 \times 10^6$ syngeneic marrow cells that had been treated with anti-theta and complement. These adult-thymectomized, irradiated, marrow-reconstituted mice (ATxBM) were used in experiments 30–60 days later. Animals that had thymic remnants revealed by necropsy at the end of each experiment were excluded.

### RESULTS

### *Lymphnode cells draining regressing sarcomas behave* in vitro *similarly to those draining progressing sarcomas*

There were two objectives here: (1) to determine whether lymphnode cells draining Moloney sarcomas late in the course of regression would recover cytolytic activity *in vitro*, and (2) if so, to compare their response to that of LNC draining progressing Moloney sarcomas. As shown in Fig. 1, regressor LNC responded similarly to progressor LNC. Peak levels of cytolytic activity, reached in both types of LNC cultures by the 4th day, were invariably higher for regressor populations, provided that the LNC cultures were initiated at the same time after tumour induction. After 4 days, cytolytic activity declined to preculture levels, usually within 8–11 days after cultures were started (data not shown).

The killing that developed in culture

FIG. 1.—Resurgence of cytolytic activity against MSC target cells and mediated by lymphocytes from popliteal and lumbar lymph nodes draining regressing (■——■) or progressing (○ – – – ○) Moloney sarcomas. Antigenically unrelated P815 mastocytoma cells were not killed in parallel experiments.

FIG. 2.—(*a*) Proliferative activity of tumour-draining LNC, as determined by assays for DNA synthesis, cell division and blast transformation, increasing to a maximum at 3 days in culture. (*b*) Percentages of T (▨) and B (□) lymphocytes determined from direct immunofluorescence analyses of LNC suspensions before and after culture. Numbers of LNC recovered at daily intervals from replicate cultures are expressed as the percent of total cells initially cultured.

FIG. 3.—(*a*) DNA synthesis and blast transformation of cultured, non-immune LNC increasing slowly to maximum levels at 5 days. At that time, low levels of cytolytic activity, expressed as the number of lytic units/$10^6$ T cells were detectable against both antigenically relevant (MSC ▧) and irrelevant (P815▨) cells. (*b*) Percentages of T (▨) and B (□) lymphocytes determined from direct immunofluorescence analyses of LNC suspensions cultured from normal, non-immune mice; numbers of LNC recovered from replicate cultures at the specified intervals are expressed as the percent of total cells initially cultured.

was antigenically specific. The MSC cell, which had been used to induce the tumours, was killed efficiently. By comparison, cultured LNC were minimally cytolytic to cells from 3 antigenically dissimilar BALB/c sarcoma cell lines.

*Resurgence of cytolytic activity* in vitro *was T-lymphocyte-dependent and associated with proliferation*

The killer cells generated in culture were T lymphocytes; cytolytic activity was abolished by treatment of cultured LNC with anti-theta serum and complement. Similar treatment with complement alone had no effect. Cytolytic activity that developed was susceptible to anti-theta treatment irrespective of whether cultures were initiated with cells taken from lymph nodes draining regressing or progressing sarcomas.

Proliferative activity, documented by: (1) uptake of $^{125}$I-IUdR into newly synthesized DNA, (2) counting of mitotic figures in cultures exposed for 3 h to vinblastine, and (3) quantification of blast cells in Giemsa-stained cytocentrifuge preparation, reached peak levels after 3 days of culture (Fig. 2(a)), one day before the peak of cytolytic activity. Cell proliferation was insufficient, however, to keep pace with cell losses that occurred *in vitro*. This fact was reflected by the steadily declining recovery values that were obtained as time in culture increased (Fig. 2(b)). While they were never as cytolytic, cultured LNC from mice with progressing sarcomas were closely similar quantitatively to cultured regressor LNC with regard to percentages of T and B lymphocytes, recovery, and proliferative activity.

While development of cytolytic activity by cultured LNC from tumour-bearing mice may have required proliferation of precursor killer cells, cell division *per se* was not sufficient to induce cytolytic activity in cultures of LNC from normal mice. Indices of proliferation on Day 5 had reached levels comparable to those obtained in 3 days using LNC from tumour-bearing mice (Fig. 3(a)). In spite of comparable levels of proliferation, the cytolytic activity measured was low (3·4 LU/$10^6$ T cells *vs* MSC targets) and relatively non-specific (2·2 LU/$10^6$ T cells *vs* P815 mastocytoma cells (Fig. 3(a)). By contrast, the cytolytic activity mediated in this same experiment by cultured regressor LNC was 40, 181 and 82 LU/$10^6$ T cell *vs* MSC cells, at 3, 4 and 5 days respectively, without detectable killing of P815 targets. Cell recoveries during the first days of culture of normal LNC were poorer than when LNC from tumoured mice were used (Fig. 3(b)). Because of this fact, normal LNC cultures were not monitored after 5 days.

*Effect of culture conditions on cytolytic activity*

The 2-ME included in cultures was not directly responsible for inducing the high levels of killing which was directed at MSC cells. This fact is shown in the Table, where results are given from an experiment in which normal and regressor LNC were cultured either with or without 2-ME. In the absence of 2-ME, cell losses for both populations were increased, and proliferative and cytolytic activities were substantially reduced. The presence of 2-ME ($5 \times 10^{-5}$M) accentuated the killing capacity for tumour-draining LNC without inducing significant levels of non-specific cytoxicity. Under identical culture conditions, 2-ME caused non-immune LNC to develop a low but significant cytolytic capability that was completely non-specific.

Because macrophages had previously been shown to inhibit the development of cytotoxicity *in vitro* (Gillespie and Russell, 1978), we examined the effect of removing the few MØ that were normally present in our LNC populations, along with other Fc-receptor-bearing types either before or after culture. LNC suspensions depleted of MØ before culture mediated levels of tumour-specific killing (43 and 55 LU/$10^6$ T cells after 2 and 4 days, respectively) that were comparable to those of LNC

TABLE.—*Effect of 2-mercaptoethanol on resurgence of cytolysis mediated by cultured lymphnode lymphocytes*

| Source of lymphocytes | 2-ME ($5\times10^{-5}$M) | % recovery of Total cells | % recovery of T cells | $^{125}$IUdR uptake (ct/min/$10^6$ cells) | Cytotoxicity* against MSC | Cytotoxicity* against P815 |
|---|---|---|---|---|---|---|
| Regressor mice† | − | 27·8 | 41·1 | 9021±535 | 13·5 | 0 |
| | + | 53·7 | 61·3 | 21,877±486 | 48·2 | 1·2 |
| Normal mice | − | 22·2 | 32·4 | 993±54 | 0 | 0 |
| | + | 63·8 | 76·0 | 46,928±1378 | 3·3 | 3·1 |

* Lytic units/$10^6$ T lymphocytes.
† 11 days after tumour induction.

FIG. 4.—After 3 days *in vitro*, $15\times10^6$ tumour-draining LNC were administered i.v. to ATxBM mice (interrupted lines) that had received an i.m. inoculation of $5\times10^3$ MSC cells 1 (a), 3 (b) or 6 (c) days previously (arrows). Tumours ceased growing and disappeared in these animals, but continued to grow progressively in groups of ATxBM mice that received either physiological saline or $15\times10^6$ similarly cultured LNC from normal, non-immune mice (solid lines).

(from the same original preparation) that were MØ-depleted *after* culture (45 and 68 LU/$10^6$ T cells, respectively).

*Cytotoxic, cultured immune LNC protected against tumour progression* in vivo

Cultured, tumour-draining LNC that were adoptively transferred to thymectomized, irradiated, marrow-reconstituted adult mice conferred protection against lethal tumour inocula. Lymphocytes were harvested from cultures at the peak of blastogenesis (Day 3) and administered i.v. to ATxBM mice ($15\times10^6$ LNC/mouse) that had received an i.m. injection of $5\times10^3$ MSC cells, 1 3 or 6 days previously. Tumour growth was monitored at 2–4-day intervals, and tumour size recorded as previously described (Russell and Cochrane, 1974). Tumour size increased roughly linearly in mice that received either physiological saline or cultured, non-immune LNC. Mice injected with cultured, tumour-draining LNC rejected their tumours completely, even with a period as long as 6 days between tumour induction and adoptive transfer (Fig. 4).

The minimum number of cultured, tumour-draining LNC required to prevent Moloney sarcoma progression in immunologically compromised hosts was determined by administering 2, 4 or $8\times10^6$ cultured immune lymphocytes 1 day after inoculation of $5\times10^3$ MSC cells. As illustrated in Fig. 5, tumour growth in mice receiving $2\times10^6$ cultured LNC was not altered significantly from that in mice injected with physiological saline (control). Mice injected with $4\times10^6$ cells all developed lethal neoplasms, but the rate of growth was slowed in comparison with that of the two former groups. Tumours never developed in mice protected with $8\times10^6$ cultured LNC.

FIG. 5.—Tumour growth monitored at 2–7-day intervals after 2 (○), 4 (□) or 8 (△) × $10^6$ cultured. tumour-draining LNC (interrupted lines) or physiological saline (solid lines) was administered i.v. to ATxBM male mice that had received an i.m. inoculation of 5 × $10^3$ cells the day before.

### DISCUSSION

Results of the experiments described here and in earlier publications (Gillespie *et al.*, 1977; Gillespie and Russell, 1978) allow 3 conclusions to be drawn about tumour-T-lymphocyte interrelationships in the Moloney system: (1) an *in vivo* control mechanism stops the production of cytolytic T lymphocytes in lymphnodes draining either regressing or progressing Moloney sarcomas; (2) after they have reached a non-cytolytic state, explantation of T lymphocytes from tumour-draining lymph nodes into culture releases them from whatever influence it is that prevents the production of killer cells *in vivo*, allowing a resurgence of cytolytic activity *in vitro*: and (3) cytolytic cells generated from non-cytolytic T lymphocytes in tumour-draining lymph nodes have powerful protective effects against tumour progression *in vivo*.

We have documented previously the functional activity *in vitro* of T lymphocytes isolated directly from regressing or progressing Moloney sarcomas, or from the lymph nodes draining these tumours (Gillespie *et al.*, 1977; Gillespie and Russell, 1978). Early in tumour development, specific cytotoxic activity was demonstrable. T lymphocytes recovered later were progressively less cytolytic, with virtual loss of this ability coinciding with either the onset of tumour progression or the disappearance of regressing sarcomas. In the experiments reported here, we confirmed an earlier observation that non-cytolytic T cells from the lymph nodes draining progressing Moloney sarcomas spontaneously regenerated their cytolytic activity when cultured *in vitro*. We have additionally shown that a similar response follows the explantation of non-cytolytic cells from lymph nodes that are draining sarcomas in the advanced stages of regression. In either case killing, which was highly specific, was shown to be lymphocyte-dependent and increased to peak levels after 4 days of culture. Increased cytolytic activity of cultured LNC was associated with marked proliferation (DNA synthesis, blast transformation, cell division), which was maximal by the 3rd culture day. Both proliferative and cytotoxic activities declined sharply after their respective peak levels were attained. Quantitatively, T lymphocyte populations from lymph nodes draining regressing Moloney sarcomas consistently reached higher peak levels of cytotoxic activity *in vitro* than populations from mice bearing progressing sarcomas. Proliferation *per se* was not responsible for the production of killer T cells, however, as unsensitized LNC cultured under the same conditions failed to develop comparable levels of cytolysis, even though they did undergo substantial proliferation *in vitro*.

These changes do not appear to be unique to the Moloney system. Several groups of investigators (Sudo and Hashimoto, 1971; DeLandazuri and Herberman, 1972; Vasudevan *et al.*, 1973; Laux and Lausch, 1974; Kamat and Henney, 1976, 1977) have observed that cytolytic activity, mediated by T lymphocytes from tumour-bearing or tumour-immune animals, was augmented significantly if they were held in culture before challenging them with target cells *in vitro*. It is not possible to determine whether the resurgence of killing in the first 4 reports was other than qualitatively similar to what we observed, since full examinations of the characteristics of the cells were not made. Kamat and Henney (1976, 1977), however, have described in detail a somewhat analogous phenomenon in an allogeneic situation. These workers concluded that 24 h of incubation *in vitro* was sufficient for the differentiation of pre-killer T cells into cytolytic effector cells. This differentiation process, which was dependent upon protein synthesis but independent of DNA synthesis or antigenic stimulation, was shown to be distinct from the antigen-driven pathway by which memory cells are specifically activated *in vitro*. This "spontaneous augmentation" of allogeneic T-cell-mediated killing was not affected by addition of alloantigen, and was greatest before and during the peak *in vivo* response. Thereafter, antigenic stimulation was required for a substantial *in vitro* response (presumably *via* memory-cell activation). By contrast, we have observed that the dramatic *in vitro* resurgence of cytotoxicity that occurred 4–6 days after the peak cytolytic activity in tumour-bearing mice was suppressed by the addition of exogenous antigen to the culture (Gillespie and Russell, 1978).

In an extension of their previous findings, Djeu *et al.* (1976) established that cooperation among T lymphocytes, complement-receptor lymphocytes and macrophages was required for spontaneous reactivation of killer cells after *in vitro* incubation (16–24 h) of non-cytolytic spleen cells from tumour-immune rats. However, these reactivated killer cells were not T lymphocytes, but were identified as complement-receptor lymphocytes.

Our results therefore may resemble more closely those obtained in a system using hapten-conjugated spleen cells instead of tumour cells as the inoculum (Starzinski-Powitz *et al.*, 1976). Specifically, cytolytic T lymphocytes were demonstrated inconsistently in lymph nodes draining inoculation sites; however, when these LNC were cultured for 48–72 h, potently cytolytic T cells developed by a proliferation-dependent pathway. Subsequent work by these investigators has demonstrated that *in vitro* differentiation of pre-killer T cells into cytolytic effector cells is blocked by a T lymphoblast that is cyclophosphamide-sensitive (Wagner *et al.*, 1976; Röllinghoff *et al.*, 1977).

The mechanism responsible for regulating the development of killer T cells *in vivo* remains unclear. To hypothesize a single pathway is, perhaps, naïve. For example, we have shown that soluble tumour antigen, macrophages, or a combination of these interfered with resurgence of cytolytic activity *in vitro* (Gillespie and Russell, 1978). These observations are consonant with a large body of evidence that both excess tumour antigen (Alexander *et al.*, 1969; Currie and Basham, 1972; reviewed in Kamo and Friedman, 1977) or macrophages (Parkhouse and Dutton, 1966; Kirchner *et al.*, 1974; Wing and Remington, 1977) can suppress antitumour responses *in vitro* and *in vivo*. Clearly, these may not be the principal factors for, as demonstrated in the experiments reported here, lytic activity diminished spontaneously after the 4th day in culture, in spite of the fact that tumour cells (*i.e.* a source of new tumour antigen) and Fc-receptor-bearing cells (including MØ) had been depleted. Loss of cytotoxic activity in association with reduced proliferative activity is a characteristic feature shared with the previously described *in vitro* anti-hapten response (Starzinski-Powitz *et al.*, 1976) and the long-term mixed-leucocyte culture system that has been investigated so thoroughly by MacDonald and his colleagues (1974 *a*, *b*; reviewed by Engers and MacDonald, 1975). In view of these considerations, and since the T lymphocyte was the predominant cell type remaining in our experiments, we are currently investigating the contribution that suppressor T cells generated *in vitro* during the proliferative phase may have to the continued production of cytolytic T cells.

The results of adoptive-transfer experiments that we conducted suggest how important continued production of killer T cells might be to the survival of tumour-bearing hosts. Cytolytic T lymphocytes, generated *in vitro* from non-cytolytic precursor cells from lymph nodes draining tumours, protected immunologically deficient mice bearing Moloney sarcomas. Sarcoma growth was slowed greatly by $4 \times 10^6$ of these cells administered i.v., and $8 \times 10^6$ afforded complete protection, even when tumours were allowed 6 days in which to become established. These results should not be interpreted to mean that T-lymphocyte-mediated killing of neoplastic cells within the tumours was the mechanism of protection. Several other possibilities must be considered. For example, in preliminary experiments (not described) draining lymphnode T lymphocytes activated *in vitro* as we have described had the capacity to activate macrophages to become non-specifically cytolytic for tumour cells. Such an indirect mechanism may ultimately be shown to be equally important to, or even more important than, the direct mediation of cytotoxicity.

Note added in proof: Schechter and Feldman (1978, *Israel J. Med. Sci.*, **14**, 131) have observed that spleen cells cultured *in vitro* from tumour (3LL)-bearing C57BL/6 mice regenerated cytolytic activity, and in Wynn-type transfer experiments significantly delayed tumour development.

We thank Mr R. G. Hoskins and Mrs A. T. McIntosh for excellent technical assistance. This is Publication No. 1438 from the Department of Immunopathology, Scripps Clinic and Research Foundation, and was supported by USPHS Grants CA-23686, AI-07007 and contract CB-44001. S.W.R. is the recipient of Research Career Development Award CA-00228.

### REFERENCES

ALEXANDER, P., BENSTED, J., DELORME, E. J., HALL, J. G. & HODGETT, J. (1969) The cellular immune responses to primary sarcomata in rats. II. Abnormal responses of nodes draining the tumour. *Proc. Roy. Soc. Lond. B*, **174**, 237.

BALDWIN, R. W., PRICE, M. R. & ROBINS, R. A. (1973) Inhibition of hepatoma-immune lymph node cell cytoxicity by tumor bearer serum and solubilized hepatoma antigen. *Int. J. Cancer*, **11**, 527.

CURRIE, G. A. & BASHAM, C. (1972). Serum mediated inhibition of the immuno-logical reactions of the patient to his own tumour: a possible role for circulating antigen. *Br. J. Cancer*, **26**, 427.

DELANDAZURI, M. O. & HERBERMAN, R. B. (1972) *In vitro* activation of cellular immune response to Gross virus-induced lymphoma. *J. Exp. Med.*, **136**, 969.

DJEU, J. V., GLASER, M., HUANG, K. Y. & HERBERMAN, R. B. (1976) Participation of three lymphoid cell types in the *in vitro* activation of cell-mediated immunity to a syngeneic Gross virus-induced lymphoma in rats. *Cell. Immunol.*, **23**, 268.

ENGERS, H. D. & MACDONALD, H. R. (1975) Generation of cytolytic T lymphocytes *in vitro*. In *Contemporary Topics in Immunobiology*. Ed. W. O. Weigle. New York: Plenum Press, V. **5**, p. 145.

FUJIMOTO, S., GREENE, M. I. & SEHON, A. H. (1976) Regulation of the immune response to tumor antigens. I. Immunosuppressor cells in tumor-bearing hosts. *J. Immunol.*, **116**, 791.

GILLESPIE, G. Y., HANSEN, C. B., HOSKINS, R. G. & RUSSELL, S. W. (1977) Inflammatory cells in solid murine neoplasms. IV. Cytolytic T lymphocytes isolated from regressing or progressing Moloney sarcomas. *J. Immunol.*, **119**, 564.

GILLESPIE, G. Y. & RUSSELL, S. W. (1978) Development and persistence of cytolytic T lymphocytes in regressing or progressing Moloney sarcomas. *Int. J. Cancer*, **21**, 94.

HOLDEN, H. T., Haskill, J. S., KIRCHNER, H. & HERBERMAN, R. B. (1976) Two functionally distinct anti-tumor effector cells isolated from primary murine sarcoma virus-induced tumors. *J. Immunol.*, **117**, 440.

KAMAT, R. & HENNEY, C. S. (1976) Studies on T cell clonal expansion. I. Suppression of killer T cell production *in vivo*. *J. Immunol.*, **115**, 1592.

KAMAT, R. & HENNEY, C. S. (1977) Studies on T cell clonal expansion. II. The *in vitro* differentiation of pre-killer and memory T cells. *J. Immunol.*, **116**, 1490.

KAMO, I. & FRIEDMAN, H. (1977) Immunosuppression and the role of suppressive factors in cancer. *Adv. Cancer Res.*, **25**, 271.

KEDAR, E., DELANDAZURI, M. O. & BONAVIDA, B. (1974) Cellular immunoadsorbents: a simplified technique for separation of lymphoid cell populations. *J. Immunol.*, **112**, 1231.

KIRCHNER, H., CHUSED, T. M., HERBERMAN, R. B., HOLDEN, H. T. & LAVRIN, D. H. (1974) Evidence of suppressor cell activity in spleens of mice bearing primary tumors induced by Maloney sarcoma virus. *J. Exp. Med.*, **139**, 1473.

KUPERMAN, O., FORTNER, G. W. & LUCAS, Z. J. (1975) Immune response to a syngeneic mammary adenocarcinoma. III. Development of memory and suppressor functions medulating cellular cytotoxity. *J. Immunol.*, **115**, 1282.

LAUX, D. & LAUSCH, R. N. (1974) Reversal of tumour mediated suppression of immune reactivity by *in vitro* incubation of spleen cells. *J. Immunol.*, **112**, 1900.

MACDONALD, H. R., ENGERS, H. D., CEROTTINI, J.-C. & BRUNNER, K. T. (1974*a*) Generation of cytotoxic T lymphocytes *in vitro*. II. Effect of repeated exposure to alloantigens on the cytotoxic activity of long-term leukocyte cultures. *J. Exp. Med.*, **140**, 718.

MACDONALD, H. R., CEROTTINI, J.-C. & BRUNNER, K. T. (1974*b*) Generation of cytotoxic T lymphocytes *in vitro*. III. Velocity sedimentation studies of the differentiation and fate of effector cells in long-term mixed leukocyte culture. *J. Exp. Med.*, **140**, 1511.

PARKHOUSE, R. M. E. & DUTTON, R. W. (1966) Inhibition of spleen cell DNA synthesis by autologous macrophages. *J. Immunol.*, **97**, 663.

PLATA, F., MACDONALD, H. R. & SORDAT, B. (1976) Studies on the distribution and origin of cytolytic T lymphocytes present in mice bearing Moloney murine sarcoma virus (MSV)-induced tumors. *Bibl. Haematol.*, **43**, 274.

RÖLLINGHOFF, M., STARZINSKI-POWITZ, A., PFIZENMAIER, K. & WAGNER, H. (1977) Cyclophosphamide-sensitive T lymphocytes suppress the *in vivo* generation of antigen-specific cytotoxic T lymphocytes. *J. Exp. Med.*, **145**, 455.

RUSSELL, S. W. & COCHRANE, C. G. (1974) The cellular events associated with regression and progression of murine (Moloney) sarcomas. *Int. J. Cancer*, **13**, 54.

RUSSELL, S. W., DOE, W. F., HASKINS, R. G. & COCHRANE, C. G. (1976*a*) Inflammatory cells in solid murine neoplasms. I. Tumor disaggregation and identification of constituent inflammatory cells. *Int. J. Cancer*, **18**, 322.

RUSSELL, S. W., GILLESPIE, G. Y., HANSEN, C. B. & COCHRANE, C. G. (1976*b*) Inflammatory cells in solid murine neoplasms. II. Cell types found throughout the course of Moloney sarcoma regression and progression. *Int. J. Cancer*, **18**, 331.

STARZINSKI-POWITZ, A., PFIZENMAIER, K., RÖLLINGHOFF, M. & WAGNER, H. (1976) *In vivo* sensitization of T cells to hapten-conjugated syngeneic structures of major histocompatibility complex. I. Effect of *in vitro* culture upon generation of cytotoxic T lymphocytes. *Eur. J. Immunol.*, **6**, 799.

SUDO, H. & HASHIMOTO, Y. (1971) Enhancement of cytotoxic activity of immune peritoneal lymphocyte cells against antigenic tumor cells after *in vitro* culture. *Gann*, **62**, 275.

TAKEI, F., LEVY, J. G. & KILBURN, D. G. (1977), Characterization of suppressor cells in mice bearing syngeneic mastocytoma. *J. Immunol.*, **118**, 412.

VASUDEVAN, D. M., BRUNNER, K. T. & CEROTTINI, J.-C. (1973) Increased cytolytic effect of immune lymphocytes in a syngeneic tumour system following simple purification procedures. *Br. J. Cancer*, **28**, (Suppl. I) 35.

WAGNER, H., STARZINSKI-POWITZ, A., PFIZENMAIER, K. & RÖLLINGHOFF, M. (1976) Regulation of T cell-mediated cytotoxic allograft responses. I. Evidence for antigen-specific suppressor T cells. *Eur. J. Immunol.*, **6**, 873.

WING, E. J. & REMINGTON, J. S. (1977). Studies on the regulation of lymphocyte reactivity by normal and activated macrophages. *Cell. Immunol.*, **30**, 108.